# Low-Level Laser Therapy for Thumb Carpometacarpal Joint Osteoarthritis: A Randomized Controlled Trial

**DOI:** 10.7759/cureus.57883

**Published:** 2024-04-09

**Authors:** Mohammad Muhibbur Rahman, Mohammad Abdus Shakoor, Nadia Ferdous, Mohammad Obaidul Alam, Shamim Farhad, A.B.M. Mehedi, Shahina Sarker, Mohammad Moyeenuzzaman

**Affiliations:** 1 Physical Medicine and Rehabilitation, Government Employee's Hospital, Dhaka, BGD; 2 Physical Medicine and Rehabilitation, Bangabandhu Sheikh Mujib Medical University (BSMMU), Dhaka, BGD; 3 Medicine, Government Employee's Hospital, Dhaka, BGD; 4 Physical Medicine and Rehabilitation, Universal Medical College and Hospital, Dhaka, BGD; 5 Physical Medicine and Rehabilitation, Savar Upazilla Health Complex, Dhaka, BGD

**Keywords:** functional capability, photobiomodulation, low-level laser therapy, osteoarthritis, first carpometacarpal joint

## Abstract

Background and aim

Low-level laser therapy (LLLT) is considered a promising non-invasive treatment option for osteoarthritis (OA). The current study aimed to evaluate the effectiveness of LLLT on patients with OA of the first carpometacarpal joint (CMC1) of the thumb.

Methods

An open-level, prospective, randomized controlled trial was conducted in the Department of Physical Medicine and Rehabilitation, Bangabandhu Sheikh Mujib Medical University (BSMMU), Dhaka, for one year. Initially, 120 patients were approached for the study. Among them, 112 eligible patients were randomly divided into two groups: the intervention group received LLLT in addition to conservative treatment, while the control group received conservative treatment alone for four weeks. Pain and functional capability (motor) improvement were assessed on a weekly follow-up basis by using various parameters such as the visual analogue scale (VAS), Ritchie articular index (tenderness scale), grip strength, key pinch strength, Dreiser functional index, and CMC1 palmer abduction. Eventually, 90 patients completed the follow-ups and were included in the analysis.

Results

The majority of patients diagnosed with CMC1 joint OA were in their fifties. At baseline, patients of both intervention and control groups were indifferent in terms of demography, pain intensity, motor responses, and duration of suffering. After four weeks of treatment, results indicated an overall improvement in both groups. However, the reduction of pain and increase in functional capability were not found statistically significant (p-value: ≥0.5).

Conclusion

LLLT with conventional treatment was not found significantly more effective enough than conventional treatment alone, but more well-designed clinical trials with larger sample sizes are needed to reach a definitive conclusion.

## Introduction

Hands are most frequently affected by the degenerative joint condition, osteoarthritis (OA). Based on different definitions of hand OA, the occurrence of hand OA as shown on imaging tests can range from 21% to 92%. However, the occurrence of symptomatic hand OA is much lower, ranging from only 3% to 16% [1]. OA of the hand affects a lot of people, and it causes them to have symptoms including pain or discomfort, stiffness, decreased mobility, and decreased grip strength. Because of this, it is difficult for individuals to use their hands and carry out the activities of daily living (ADL) [2]. The thumb's carpometacarpal (CMC1) joint is a common site for Hand OA, affecting as many as 15% of the population over the age of 30 [1,3]. Frequent pain and weakness, especially while gripping and pinching, are the primary manifestations of CMC1 OA [4].

For OA of CMC1, there are a variety of alternatives for treatment, including conservative treatment modalities (modification of daily activities, non-steroidal anti-inflammatory drugs (NSAIDs), intra-articular steroid injections, splinting, and physical therapy) and surgical modalities (arthroplasties and arthrodesis, etc.). However, each has advantages and inherent limitations and complications [5-7]. Low-level laser therapy (LLLT), also known as photobiomodulation, has been suggested as a potential treatment for acute and chronic pain in OA patients. It has been found to stimulate cell growth, improve blood flow, promote the development of blood vessels, and increase collagen production. These effects help reduce inflammation and degeneration in soft tissues such as fascia, ligaments, and muscles [8]. Studies have shown that LLLT can treat pain caused by a variety of conditions including epicondylitis lateralis (tennis elbow), carpal tunnel syndrome, OA knee, and rheumatoid arthritis [9-11]. Still now, the effectiveness of LLLT for the treatment of hand OA remains contentious.

Studies mostly explored the effect of LLLT on knee OA and reported favorable outcomes, while a few studied the effect on hand OA [12]. Brosseau et al. (2005) conducted a randomized controlled study (RCT) on people with OA of the hand and found that LLLT was no more effective than a placebo at reducing pain, morning stiffness, or improving functional status [13]. However, in another RCT, Paolillo et al. (2015) showed that a device combining LLLT and ultrasound significantly reduced pain in patients with hand OA [14]. Overall, the effects of LLLT on hand OA are still up for debate and require more research.

Moreover, nowadays, LLLT treatment has become available and popular among Bangladeshi patients suffering from musculoskeletal disorders. Considering the sufferings of the patient due to OA of the CMC1 joint and limited and controversial research evidence, we planned to conduct research to examine the effectiveness of LLLT for reducing pain and increasing functional capability in patients with CMC1 OA.

## Materials and methods

An open-level, RCT was conducted among patients presenting with thumb OA at the Department of Physical Medicine and Rehabilitation of Bangabandhu Sheikh Mujib Medical University (BSMMU), Dhaka, Bangladesh, from July 1st, 2014 to June 30th, 2015. This study complied with the principles of the Declaration of Helsinki regarding investigations in humans and was approved by the BSMMU Institutional Review Board (No. BSMMU/2014/8056).

Selection of the study subjects

This study included patients of both sexes aged 40 to 75 years who met the American College of Rheumatology diagnostic criteria for OA of the hand, thumb pain due to chronic non-inflammatory causes, pain duration greater than one month, and a stable level of ADL [15]. Patients with a history of neurologic disease or other disabling conditions, who had undergone previous treatment for their hand issue within the past six months (such as an intraarticular joint injection), had fractures or serious hand injuries, had undergone previous thumb surgery, were pregnant women, or had a history of light sensitivity or a skin lesion were excluded from the study. The aims and objectives of the study were discussed with the patients, and informed written consent was obtained. Initially, 120 patients were approached to participate in the study based on inclusion and exclusion criteria, but eight patients declined. Eventually, 112 patients were included in the study. Participant demographic (age, gender, etc.) data, duration of illness, hand dominance, most affected site of CMC1, presence of potential risk factors associated with CMC1 OA, previous treatment or therapy (medication, injection, splint, rehabilitation, and surgery), baseline investigations, radiographic evidence of hand OA, etc., were collected.

Allocation of treatment/intervention

Participants were assigned randomly to either the intervention group or the control group. In the intervention group, 64 patients were treated with LLLT along with conventional treatment, including a thumb splint daily at night, ADL advice (joint protection technique), and Paracetamol 665 mg twice daily. In the control group, 48 patients were given conservative treatment (analgesics, a night splint, and joint protective techniques).

Treatment (LLLT) description

The intervention group's LLLT device (Endolaser 476 (Enraf-Nonius), Delft, Holland) was of the solid type. The active medium of the device was Ga-As-Al, with a wavelength of 830 nm and a pulsed maximum output power of 50 mW. The repetition frequency of the pulse is 300 Hz. A single hand-held LASER probe delivered the LASER beam. This LASER device delivered a maximum energy density of 4 J/cm2 per point irradiated in modulated mode. For four weeks, treatment was provided on alternate days (three days) per week [11]. LLLT was delivered through static light and probe contact with the skin. The probe was positioned at a 90° angle to the joint. Before each session, the probe tip and skin were cleansed with an alcohol wipe. To safeguard their eyes, both the LASER applicator and the receiving patient donned protective eyewear. 

Outcome assessment

**Figure 1:**
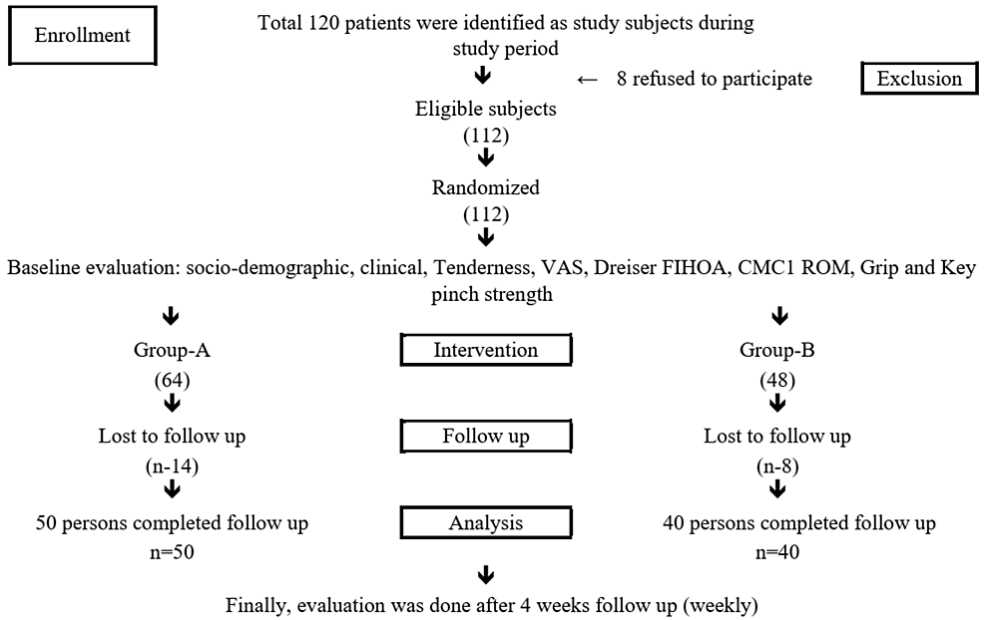
Study flow chart VAS: visual analogue scale; FIHOA: functional index of hand osteoarthritis; CMC1 ROM: first carpometacarpal joint range of motion

Following the completion of the scheduled therapy, patients in both groups were followed up on a weekly basis for four weeks, and the outcomes were correctly noted in the datasheet. Pain assessment of the patients was done during follow-up visits by the visual analogue scale (VAS), Ritchie articular index (tenderness scale), and motor performance was assessed by grip strength by JAMAR hand dynamometer, key pinch strength by JAMAR pinch gauze, Dreiser's functional index of hand osteoarthritis (FIHOA) by answering a four-point verbal scale on physical function, and CMC1 palmer abduction by Goniometer [16-18]. Eventually, 14 patients from the intervention group and eight patients from the control group were lost during follow-up. In the end, 50 patients from the intervention group and 40 from the control group were included in the analysis. A flow diagram of the subject recruitment process for the study is provided in Figure 1.

Statistical analysis

The statistical analyses were carried out using the Statistical Package for Social Science (SPSS), Version 23 (IBM Inc., Armonk, New York). Continuous data were summarized using means and standard deviations. The data's normality distribution was assessed using the Shapiro-Wilk test. Independent sample t-tests/Mann-Whitney U test was done to determine the difference of continuous variable between groups at baseline and four weeks following treatment. A paired t-test/Wilcoxon signed-rank test was done to determine the changes from baseline to four weeks following treatment. To determine the association between categorical variables, Chi-square tests/Fisher’s exact test was done. A p-value of <0.05 was considered to indicate statistical significance.

## Results

At baseline, both groups were statistically similar or indifferent in terms of age, sex, duration of symptom, VAS score, tenderness and tenderness scale, grip strength (kg), key pinch score (kg), Dreiser’s FIHOA, and CMC1 palmer abduction (p-value: >0.05) (Table 1).

**Table 1:** Comparison of baseline characteristics of patients of both groups (N=90) Data were presented as mean±SD and frequency (%). Independent sample t-test, Mann-Whitney U test, and Chi-square test were done. FIHOA: functional index for hand osteoarthritis; VAS: visual analogue scale; CMC1: first carpometacarpal joint; SD: standard deviation

Baseline characteristics			Intervention group (n=50)	Control group (n=40)	P-value
Age (in years) (mean±SD)			56.4±11.7	56.8±9.7	0.89
Gender		Male	19 (38.0)	16 (40.0)	0.84
		Female	31 (62.0)	24 (60.0)	
Height (cm)			156.3±4.9	157.1±4.2	0.72
Weight (kg)			69.1±8.7	68.8±9.9	0.73
Duration of symptoms (months) (mean±SD)			18.1±25.4	17.1±26.2	0.88
Pain assessment	VAS score		7.8±1.3	7.4±1.1	0.17
	Tenderness		49 (98.0)	38 (95.0)	0.58
	Tenderness scale (mean±SD)		2.8±0.6	2.6±0.7	0.05
Motor performance assessment	Grip strength (kg)		19.4±0.8	20.1±1.5	0.10
	Key pinch score (kg)		3.6±0.7	3.7±0.6	0.24
	Dreiser’s FIHOA		14.4±3.2	14.2±2.9	0.67
	CMC1 palmer abduction		33.3±2.9	32.7±2.8	0.36

After four weeks following the treatment assigned, no significant difference in terms of pain was observed among groups. Similarly, no significant variation in motor performance was observed among groups determined by grip strength (kg), Dreiser’s FIHOA, key pinch score (kg), and CMC1 palmer abduction (p-value: >0.05) (Table 2).

**Table 2:** Difference in clinical variables on follow-up (four weeks) after treatment between groups Data were presented as mean±SD. Independent sample t-test and Mann-Whitney U test were done. FIHOA: functional index for hand osteoarthritis; VAS: visual analogue scale; CMC1: first carpometacarpal joint; SD: standard deviation

Variables		Post-treatment follow-up (4 week)		P-value
		Intervention group (n=50)	Control group (n=40)	
Pain assessment	VAS score	7.6±1.3	7.3±1.1	0.11
	Tenderness scale	2.7±0.8	2.5±0.8	0.23
Motor performance assessment	Grip strength (kg)	19.9±1.6	20.4±1.6	0.18
	Key pinch score (kg)	3.8±0.7	3.8±0.6	0.53
	Dreiser’s FIHOA	14.1±3.0	13.9±2.7	0.10
	CMC1 palmer abduction	33.5±2.7	33.0±2.6	0.38

Following treatment, the VAS score, tenderness score, and Dreiser’s FIHOA reduced insignificantly in the intervention group (p-value: >0.05). Grip strength (kg), key pinch score (kg), and CMC1 palmer abduction increased insignificantly from baseline to follow-up at four weeks in the intervention group (p-value: >0.05). Similar insignificant decrease of the VAS score, tenderness score, and Dreiser’s FIHOA following treatment at four weeks of follow-up and insignificant increase of grip strength (kg), key pinch score (kg), and CMC1 palmer abduction from baseline were observed in the control group (p-value: >0.05) (Table 3).

**Table 3:** Changes in clinical variables from baseline to follow-ups after treatment (four weeks) Data were presented as mean± SD. Paired t-test and Wilcoxon signed-rank test were done. FIHOA: functional index for hand osteoarthritis; VAS: visual analogue scale; CMC1: first carpometacarpal join; SD: standard deviation

Follow-up after treatment		Variables	Baseline (Week 0)	Post-treatment follow-up (Week 4)	P-value
Intervention group (n=50)	Pain assessment	VAS score	7.8±1.3	7.6±1.3	0.07
		Tenderness scale	2.8±0.6	2.7±0.8	0.09
	Motor performance assessment	Grip strength (kg)	19.4±0.8	19.9±1.6	0.35
		Key pinch score (kg)	3.6±0.7	3.8±0.7	0.10
		Dreiser’s FIHOA	14.4±3.2	14.1±3.0	0.06
		CMC1 palmer abduction	33.3±2.9	33.5±2.7	0.06
Control group (n=40)	Pain assessment	VAS score	7.4±1.1	7.3±1.1	0.08
		Tenderness scale	2.6± 0.7	2.5±0.8	0.37
	Motor performance assessment	Grip strength (kg)	20.1±1.5	20.4±1.6	0.08
		Key pinch score (kg)	3.7±0.5	3.8±0.6	0.12
		Dreiser’s FIHOA	14.2±2.9	13.9±2.7	0.64
		CMC1 palmer abduction	32.7±2.8	33.0±2.6	0.08

**Figure 2:**
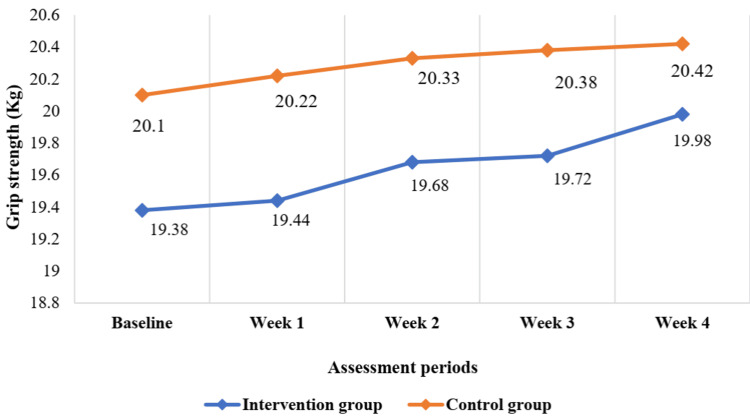
Changes in grip strength from baseline to follow-up after treatment up to four weeks

**Figure 3:**
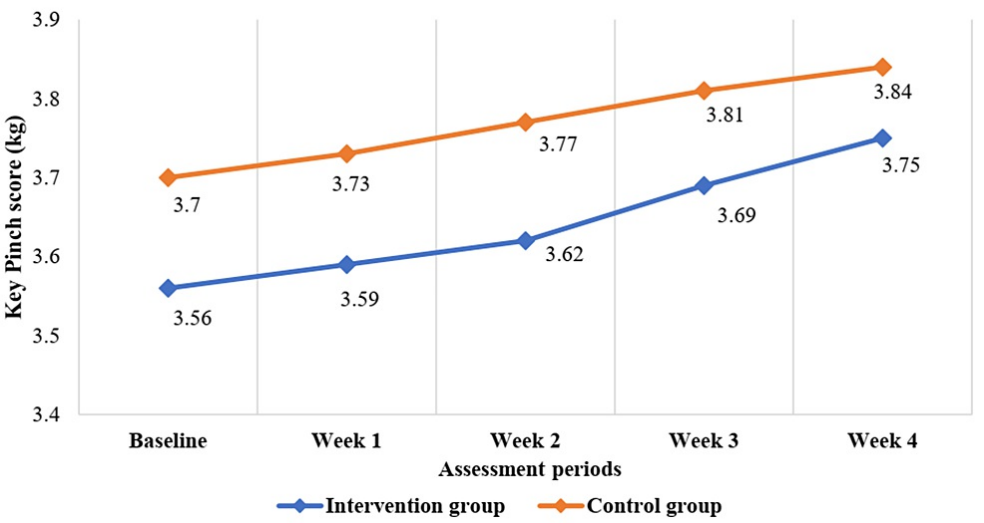
Changes in key pinch score from baseline to follow-up after treatment up to four weeks

**Figure 4:**
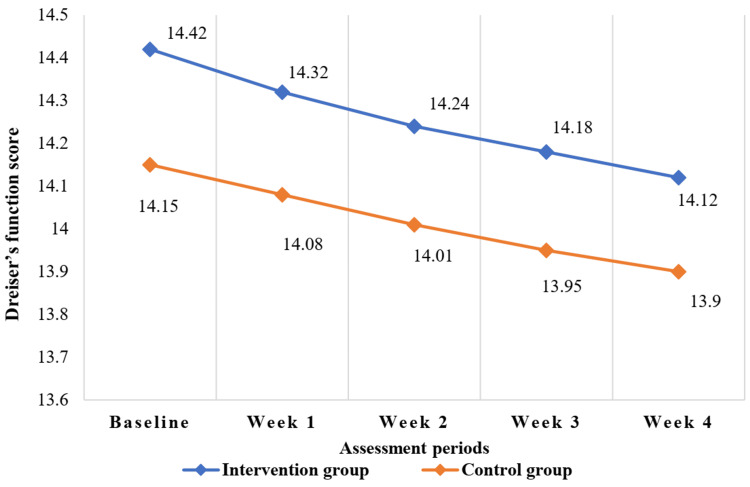
Changes in Dreiser’s function score from baseline to follow-up after treatment up to four weeks

The grip strength (kg) and key pinch score (kg) gradually increased and the Dreiser’s function score gradually decreased, but this improvement of motor function was not significant from baseline up to follow-up (four weeks) after being treated (Figure 2, Figure 3, and Figure 4 and Table 3). 

## Discussion

OA of the hand is currently treated conservatively with oral or topically applied NSAIDs, opioid analgesics, and hand rehabilitation physiotherapy [19]. LLLT has been documented as a safe, non-invasive, effective, and successful treatment method for reducing pain and inflammation, as well as improving joint mobility in individuals with Hand OA [20]. This study was planned to evaluate the effectiveness of LLLT in reducing pain and improvement of motor response/functional capability in thumb carpometacarpal joint OA. Nevertheless, this study did not find any enhancement in functional capacity (such as grip strength, key pinch strength, Dreiser's FIHOA, and CMC1 range of motion (ROM)) or pain reduction when LLLT was used in combination with conservative treatment. The results indicate that the addition of LLLT did not provide any additional benefits compared to conservative treatment alone.

In general, the risk of OA increases with advancing age, and the percentage of people who have OA climbs steadily after the age of 50 [21]. In this study, the majority of the patients suffering from thumb carpometacarpal joint OA were of the 51-60 years age group, and the mean±SD of study participants was 56.6±8.7 years. Studies conducted by Jain et al., (2016) and Bani et al. (2013) also reported the mean age similar to our study [22,23]. The mean duration of symptoms of our study population is nearly two years (17.2±28.4 months). Jain et al. (2016) got 75% of the study population with symptoms of less than one year whereas Sillem et al.(2011) found their study subjects with a mean duration of 2.99 years [22,24].

At baseline, the mean VAS score of the study population was 7.62±1.22, which is similar to another study [13,25-27]. The baseline mean Ritchie tenderness scale was 2.5±0.36. The mean (±SD) changes of VAS score and tenderness scale from week 0 (Baseline) to follow-up at four weeks in both the intervention and control group showed a tendency to decrease pain but this reduction of pain was not significantly different. Brosseau et al. (2005) reported similar findings of LLLT not being able to reduce pain effectively, whereas Paolillo et al. (2015) reported a significant reduction of pain when they used a device combining LLLT and ultrasound for the treatment of patients with hand OA [13,14]. The synergistic effect of ultrasound might be the reason for the reduction of pain.

In this study, all functional outcome variables (grip strength, key pinch strength, Dreiser’s FIHOA, and CMC1 palmer abduction) improved gradually following treatment, but this improvement was minimal in both the intervention and control groups. In this study, there was a little inclination of Dreiser’s FIHOA and an increase in hand grip and key pinch strength, but this was not enough to create any significant changes in the overall functional outcome of the hand at the last follow-up, and we did not find that individuals in this study with OA of the carpometacarpal joint of the thumb benefited significantly from LLLT at 830 nm, 50 mW, and the 4J dose used in this study. In the study conducted by Brosseau et al. (2005), where patients received LLLT (n=42) or sham LLLT (n=46), they reported similar non-significant improvements in pain relief, strength changes, and functional status for LLLT versus placebo [13]. According to a meta-analysis of RCTs conducted by Brosseau et al.(2000), no effect on pain, joint tenderness, joint mobility, and strength was observed [11].

In terms of effectiveness, LLLT appears to be joint- and disease-specific. It appears that LLLT is more beneficial in the treatment of RA, an inflammatory disease, than it is for OA [11,13]. Perhaps finger joints require a special kind of LLLT application since they are more intricate, innervated, and frequently used in ADL and repetitive isometric movements. Several factors influence how well LLLT works, including the wavelength used, the depth of penetration, the dose delivered, the length of time the therapy is applied, the amount of power delivered, the frequency of the pulses, and the treatment protocol itself [28]. These above-mentioned factors might be the reasons for not achieving significant improvement in the outcome. However, based on these results and discussion, the researchers suggested that low-power LASERs should not be applied as routine treatment before more documented scientific evidence of beneficial effects is available. 

Limitations

This study only assessed short-term outcomes in a small number of patients. Blinding was not utilized in this study, which may perhaps lead to biases. Furthermore, due to the implementation of combined LLLT with conservative treatment, we were unable to assess the individual effects of LLLT alone. The long-term effects of LLLT for hand OA are yet studied. Future studies should look into the long-term effects of LLLT on hand OA utilizing a larger sample and a variety of therapeutic applications (dosage, treatment duration, etc.).

## Conclusions

In patients with CMC1 joint OA, low-intensity laser therapy along with conservative treatment was not found to be more effective than conservative treatment alone in reducing pain and tenderness and improving the motor response (functional capability). Hence, it is not advisable to regularly employ it in clinical settings for the treatment of CMC1 joint OA until further scientific proof of its therapeutic efficacy is established.
